# Cost-effectiveness analysis of liraglutide versus sitagliptin or exenatide in patients with inadequately controlled Type 2 diabetes on oral antidiabetic drugs in Greece

**DOI:** 10.1186/1472-6963-14-419

**Published:** 2014-09-22

**Authors:** Charalampos Tzanetakos, Andreas Melidonis, Christos Verras, Georgia Kourlaba, Nikos Maniadakis

**Affiliations:** Department of Health Services Organisation and Management, National School of Public Health, 196 Alexandras Avenue, 11521 Athens, Greece; Diabetes Center, Tzanio General Hospital, Piraeus, Greece; Collaborative Center for Clinical Epidemiology and Outcomes Research (CLEO), “Aghia Sophia” Children’s Hospital, Athens, Greece

**Keywords:** Cost-effectiveness, Liraglutide, Type 2 diabetes, Greece

## Abstract

**Background:**

To evaluate the long-term cost-effectiveness of liraglutide versus sitagliptin or exenatide, added to oral antidiabetic drug mono- or combination therapy respectively, in patients with Type 2 diabetes in Greece.

**Methods:**

The CORE Diabetes Model, a validated computer simulation model, was adapted to the Greek healthcare setting. Patient and intervention effects data were gathered from a clinical trial comparing liraglutide 1.2 mg once daily vs. sitagliptin 100 mg once daily, both combined with metformin, and a clinical trial comparing liraglutide 1.8 mg once daily vs. exenatide 10 μg twice daily, both as add-on to metformin, glimepiride or both. Direct costs were reported in 2013 Euros and calculated based on published and local sources. All future outcomes were discounted at 3.5% per annum, and the analysis was conducted from the perspective of a third-party payer in Greece.

**Results:**

Over a patient’s lifetime, treatment with liraglutide 1.2 mg vs. sitagliptin drove a mean increase in discounted life expectancy of 0.13 (SD 0.23) years and in discounted quality-adjusted life expectancy of 0.19 (0.16) quality-adjusted life years (QALYs), whereas therapy with liraglutide 1.8 mg vs. exenatide yielded increases of 0.14 (0.23) years and 0.19 (0.16) QALYs respectively. As regards lifetime direct costs, liraglutide 1.2 mg resulted in greater costs of €2797 (€1468) versus sitagliptin, and so did liraglutide 1.8 mg compared with exenatide (€1302 [€1492]). Liraglutide 1.2 and 1.8 mg doses were associated with incremental cost effectiveness ratios of €15101 and €6818 per QALY gained, respectively.

**Conclusions:**

Liraglutide is likely to be a cost-effective option for the treatment of Type 2 diabetes in a Greek setting.

## Background

Type 2 diabetes mellitus (T2DM) is a complex multifactorial chronic progressive disease that is spread worldwide over the past three decades, making it a public health menace [1]. Accounting for approximately 90% of all diabetes cases, T2DM is shown to be associated with increased morbidity and mortality resulting in considerable socioeconomic implications for national healthcare systems [2]. In Greece, following the prevalence patterns of other developed countries, the prevalence of T2DM among adults was estimated at 7.6% in men and 5.9% in women [3]. According to International Diabetes Federation (IDF), the diabetes-related deaths among Greek patients aged between 20–79 years old mounted to 4906 in 2013 [4]. Furthermore, the average annual cost of treatment for a patient with T2DM in Greece was recently gauged at €1297.30 and could rise up to €2889 considering the cost of hospitalizations due to disease micro- and macrovascular complications (2.3 billion Euros or 12% of total healthcare expenditure) [5].

Current treatment recommendations advocate the use of lifestyle interventions (diet and exercise) and metformin as first-line therapy, with the subsequent stepwise additions of an oral agent (sulphonylurea, thiazolidinedione or dipeptidyl peptidase-4 [DPP-4] inhibitor), a glucagon-like peptide 1 (GLP-1) receptor agonist or an insulin (usually basal) [6, 7]. Second and third-line drug choices are based on patient and drug characteristics, with the overarching goal of improving glycaemic control while minimizing side effects [6].

In light of drug characteristics, head-to-head clinical trial data from large, controlled studies have displayed the higher or comparable safety and efficacy of liraglutide, a longer-acting GLP-1 analogue, in terms of glycated haemoglobin (HbA1c) reduction, reductions in body weight, and the drug’s low hypoglycaemic event rates compared with other anti-hyperglycaemic agents from various antidiabetic classes such as sitagliptin, exenatide and basal insulin [8]. Hence, the multifactorial clinical profile of liraglutide has been well established and, makes it a good candidate for the treatment of patients with T2DM failing first-line antihyperglycaemic therapy based on the current disease management guidelines [6].

Nonetheless, an antihyperglycaemic treatment may be an effective and safe option for T2DM, but it also imposes a tangible cost to the healthcare payers. The balance of treatment efficacy and costs should be examined to maximize value for money in healthcare spending. The aim of this study was to investigate the cost-effectiveness of liraglutide versus sitagliptin or exenatide respectively, as adjunct treatments to oral antidiabetic drug (OAD) therapy, in patients with T2DM failing first-line OAD therapy in a Greek setting.

## Methods

### Model description

The present cost-effectiveness analysis was conducted using the CORE Diabetes Model (CDM) in order to project the long-term clinical and cost outcomes associated with liraglutide and its comparators. A detailed description of the model’s design and operational features has been published elsewhere [9, 10]. The CDM is an Internet-based computer simulation model developed to determine the long-term health outcomes (life expectancy, quality-adjusted life expectancy, cumulative incidences of complications) and economic consequences (annual and cumulative costs per patient, costs associated with complications and treatment), as well as incremental cost-effectiveness ratios (ICER) of interventions in type 1 and type 2 diabetes. The model is a validated [9] non-product-specific, diabetes policy analysis tool that performs real-time simulations taking into account the baseline population characteristics, history of complications, various screening and treatment strategies for micro-vascular complications, treatment strategies for end-stage complications, as well as other multi-factorial interventions. Disease progression relies upon a series of inter-dependent Markov sub-models that simulate progression of disease related complications (angina, myocardial infarction, congestive heart failure, stroke, peripheral vascular disease, diabetic retinopathy, macula oedema, cataract, hypoglycaemia, ketoacidosis, nephropathy and end-stage renal disease, neuropathy, foot ulcer, amputation, depression, and non-specific mortality). Each sub-model uses time, state and diabetes type-dependent probabilities derived from published sources and utilizes tracker variables to overcome the memory-less properties of standard Markov models, allowing interconnectivity and interaction between individual complication sub-models.

### Simulated cohorts and treatment effects

For the comparison of liraglutide versus sitagliptin 100 mg once daily, both as add-on to metformin monotherapy, a patient cohort was defined with baseline demographics, baseline complications and physiological parameters representative of the patients enrolled in the respective treatment arms of the randomized head-to-head clinical trial (1860 Study Group) after being poorly controlled with metformin alone [11]. The chosen dose for liraglutide was 1.2 mg once daily following common clinical practice in Greece (standard dose). As regards the treatment comparison of liraglutide versus exenatide, baseline patient cohort characteristics were elicited from the Liraglutide Effect and Action in Diabetes 6 (LEAD 6) randomized head-to-head clinical trial in which patients, inadequately controlled on metformin, sulfonylurea, or both, were assigned to receive additional liraglutide 1.8 mg once daily or exenatide 10 μg BID [12]. Note the dose-escalation of liraglutide from 1.2 mg to 1.8 mg was preserved in the model analysis since no clinical data was available for 1.2 mg dose. A subgroup of patients (65%) was assumed to take also glimepiride in conjunction with metformin. Furthermore, a cohort reflective of typical Greek patients was considered and inserted in the sensitivity analysis of liraglutide versus sitagliptin or exenatide, with data extracted from Liatis et al. study [13] and other local sources (Table 1). Where input data were missing, data from 1860 clinical trial were encompassed. All simulated cohorts are shown in Table 1.

**Table 1:** **Baseline patients’ characteristics of 1860, LEAD-6 and Greek simulated cohorts**

	1860	LEAD-6	Greek ^*^
**Patient demographics**			
Baseline age (years)	55.3 (9.2)	56.7 (10.3)	64.5 (10.5)
Duration of diabetes (years)	6 (5.1)	8.2 (6)	10.4 (8.3)
Percentage male	52.9%	51.9%	51.5%
**Baseline risk factors**			
HbA1c (%)	8.4 (0.8)	8.2 (1.0)	8.2 (1.8)
Systolic blood pressure (mmHg)	132.2 (14.5)	133.0 (16.6)	139.8 (19.3)
Total cholesterol (mmol/l)	4.79 (0.38)	4.7 (0.95)	5.5 (−)
HDL (mmol/l)	1.16 (0.31)	1.2 (0.3)	1.2 (0.3)
LDL (mmol/l)	2.65 (0.82)	2.95 (0.8)	3.7 (1.0)
Triglycerides (mmol/l)	2.38 (2.22)	2.15 (1.35)	1.9 (1.4)
Body mass index (kg/m^2^)	32.8 (5.2)	32.9 (5.7)	30.4 (5.3)
Proportion smoker^¶^	36.0%	36.0%	36.0%
Cigarettes per day^¶^	18.7	18.7	18.7
Alcohol consumption (Oz/week)^¶^	15.91	15.91	15.91
**Racial characteristics**			
Proportion White	90%	80.6%	100%
Proportion Black	7.5%	5.5%	0%
Proportion Hispanic	0%	12.4%	0%
Proportion Native American	0.5%	1.3%	0%
Proportion Asian/Pacific Islander	2%	0.2%	0%
**Baseline CVD complications**			
Proportion with myocardial infarction	2.7%	2.6%	12.8%
Proportion with angina	1.8%	1.9%	5.0%
Proportion with peripheral vascular disease	0.9%	0.6%	5.6%
Proportion with stroke	0.8%	0.2%	8.1%
Proportion with congestive heart failure	0.4%	1.5%	4.2%
Proportion with atrial fibrillation	1.5%	1.5%	9.1%
Proportion with left ventricular hypertrophy	0.2%	0.2%	3.1%
**Baseline renal complications**			
Proportion with microalbuminuria	1.1%	1.1%	1.1%
Proportion with gross proteinuria	0.2%	0.2%	0.2%
Proportion with end-stage renal disease	0.4%	0.6%	0.4%
**Baseline retinopathy complications**			
Proportion with BDR	2.7%	1.1%	25%
Proportion with PDR	0.2%	0.2%	4%
Proportion with severe vision loss	0.4%	0.6%	4.5%
**Baseline macular edema**			
Proportion with macular edema	1.1%	0.2%	1.1%
**Baseline cataract**			
Proportion with cataract	1.7%	6.9%	1.7%
**Baseline foot ulcer complications**			
Prop. uninfected ulcer	0.6%	0.2%	0.6%
Prop. infected ulcer	0.3%	0.0%	0.3%
Prop. healed ulcer	0.0%	0.0%	0.0%
Prop. history of amputation	0.0%	0.2%	0.0%
**Baseline neuropathy**			
Proportion with neuropathy	11.6%	7.8%	14.7%
**Baseline depression**			
Proportion with depression^†^	0%	0%	9%

Data are mean (SD). *In the Greek cohort, all demographic and physiological parameters were retrieved from S. Liatis et al. study [13], while the renal, foot ulcer, macular oedema and cataract complications values were assumed to be the same as for 1860 cohort. All other diabetes-related complications were taken from National School of Public health (data on file) and racial characteristics from Hellenic Statistical Authority (http://www.statistics.gr). ^¶^Considering all cohorts, the smoking status and number of cigarettes per day were elicited from National School of Public health, Healthcare map 2011 (data on file), whereas the alcohol consumption estimate from S. Liatis et al. study [13]. ^†^Proportion with depression assumed to be 0 in 1860 and LEAD-6 cohorts. Liraglutide Effect and Action in Diabetes 6 = LEAD-6; HbA1c = glycated haemoglobin; HDL = high-density lipoprotein; LDL = low-density lipoprotein; CVD = cardiovascular disease; BDR = background diabetic retinopathy; PDR = proliferative diabetic retinopathy.

Treatment effects for both simulations of liraglutide vs. sitagliptin and liraglutide vs. exenatide were derived directly from the aforementioned clinical trials. These effects were applied in the first year of the modelling simulation, while their long-term progression was modelled by relying upon published studies incorporated in the CDM [10] and assumptions. A summary of treatment effects and their long-term progression is given in Table 2. Regarding the assumptions, HbA1c effect was assumed to be sustained over model simulations for both treatment arms. Duration of incretin-based therapies was set to 5 years, after which basal insulin therapy was initiated in an attempt to replicate clinical practice (OADs therapy was maintained). Further, it was assumed that body mass index (BMI) was affected during the treatment duration and returned to baseline level following treatment switching. Major and minor hypoglycaemic rates were based on 1860 and LEAD-6 trials [11, 12] for the first 5 years of simulation, and on LEAD-5 trial [14] for the remainder of model duration.

**Table 2:** **Summary of treatment effects: 1860 and LEAD-6 trials & long-term progression approach**

Risk Factor	1860 clinical trial ^*^				LEAD-6 clinical trial ^¶^				Long-term progression
	Liraglutide (1.2 mg)		Sitagliptin (100 mg)		Liraglutide (1.8 mg)		Exenatide BID (10 μg)		All comparators
	Mean	SD	Mean	SD	Mean	SD	Mean	SD	
Change from baseline in HbA1c (%)	−1.24	1.04	−0.90	1.04	−1.12	1.22	−0.79	1.22	No HbA1c creep assumption
Change from baseline in SBP (mm Hg)	−0.55	13.23	−0.94	13.17	−2.51	17.55	−2.00	17.93	UKPDS progression
Change from baseline in total cholesterol (mmol/l)	−0.03	0.82	−0.02	0.80	−0.20	1.02	−0.09	1.01	Framingham progression
Change from baseline in LDL (mmol/l)	0.08	0.69	0.13	0.68	−0.44	0.84	−0.40	0.84	Framingham progression
Change from baseline in HDL (mmol/l)	0.00	0.17	0.00	0.17	−0.04	0.23	−0.05	0.23	Framingham progression
Change from baseline in triglycerides (mmol/l)	−0.19	1.42	−0.40	1.38	−0.41	1.50	−0.23	1.48	Framingham progression
Change from baseline in BMI (kg/m^2^)	−1.00	0.08	−0.34	0.08	−1.15	1.35	−1.02	1.47	Constant after returning to baseline
Major hypoglycaemia (events per 100 patient-years)	1.00		0.00		0.00		2.00		0.00^#^
Minor hypoglycaemia (events per 100 patient-years)	17.80		10.60		193.20		260.00		128.70^#^

*P between treatment arms < 0.001 for change in HbA1c and BMI. ^¶^P between treatment arms < 0.0001 for change in HbA1c and P < 0.05 for change in triglycerides and minor hypoglycaemic events. ^#^Long-term hypoglycaemic event rates were based on the Liraglutide Effect and Action in Diabetes 5 (LEAD-5) study [14]. HbA1c = glycated haemoglobin; BMI = body mass index; LDL = low-density lipoprotein; HDL = high density lipoprotein; SBP = Systolic blood pressure; UKPDS = United Kingdom Prospective Diabetes Study; SD = standard deviation.

### Costs and utilities

The present analysis was conducted from a third-party payer perspective (National Organisation for Healthcare Services Provision [EOPYY]) and only direct healthcare costs were included in the model (Table 3). The daily drug costs were calculated by combining the daily dose of antihyperglycaemic agents, as retrieved from the corresponding clinical trials and validated by two authorized local diabetologist experts reflecting also current medical practice in Greece, with the drug unit costs provided by the most recent price bulletin issued by the Greek Ministry of Health [15] in conjunction with the internal reference price system attached to the latest published positive drug list. More specifically, the drug unit costs were based on the social security reimbursement prices minus the patient co-payment, a rebate of 9% imposed upon manufacturers to get into the positive drug list and another rebate based on volume which can be up to 8% depending on quarterly sales (law 4052/2012, Government Gazette). Finally, a conservative 5% volume-related rebate was considered on top of the 9%. Apart from antihyperglycaemic medication costs, the direct healthcare costs also reflected and encapsulated thoroughly the resource consumption incurred for the management of patients with diabetes and their developed complications. In particular, costs associated with patients’ hospitalization, outpatient visits, screening, concomitant medication, laboratory tests and management of hypoglycaemic events were considered (Table 3). All cost data were reported in 2013 values (€). Data for resource utilization for the management of T2DM and its complications were retrieved from diabetologist experts’ opinion. In addition, where available, cost of complications was extracted directly from published studies or other local sources [16–21] and cost data, not available in 2013 values, were inflated using the corresponding health inflation rates reported by the National Statistical Service [22]. Moreover, unit price data were obtained from the “Government Gazette”, as well as from data on file maintained by the National School of Public Health. Health state utility and event disutility values were the same as for an older cost-effectiveness analysis of liraglutide versus sitagliptin in the UK setting [23].

**Table 3:** **Health state, event, drug acquisition and consumable costs used in the analysis, expressed in 2013 Euros (€)**

Cost description	Costs (€)	Reference
**Management costs**		
Annual cost of statins	147.97	Experts opinion & Drug price bulletin [15]
Annual cost of aspirin	14.41	Experts opinion & Drug price bulletin [15]
Annual cost of ACE inhibitors	78.32	Experts opinion & Drug price bulletin [15]
Annual cost of screening for microalbuminuria	8.66	Government Gazette*
Annual cost of screening for gross proteinuria	8.66	Government Gazette*
Annual cost of stopping ACEIs due to side effects	15.00	Experts opinion
Annual cost of eye screening	10.00	Experts opinion & Government Gazette^¶^
Annual cost of foot screening program	10.00	Experts opinion & Government Gazette^¶^
Annual cost of non-standard ulcer treatment	0.00	This is a patient’s expenditure
Annual cost of anti-depression treatment	154.50	Experts opinion & Drug price bulletin [15]
Annual cost of screening for depression	10.00	Experts opinion & Government Gazette^¶^
**Direct costs of cardiovascular complications**		
Myocardial infarction, 1st year of event	6000	G. Kourlaba et al. [19]
Myocardial infarction, each subsequent year	1964	G. Kourlaba et al. [19]
Angina, 1st year of event	3613	National School of Public Health (data on file)
Angina, each subsequent year	1197	National School of Public Health (data on file)
Congestive heart failure, year of onset	3910	National School of Public Health (data on file)
Congestive heart failure, each subsequent year	1398	National School of Public Health (data on file)
Stroke, 1st year of event	4406	G. Kourlaba et al. [19]
Stroke, each subsequent year	1976	G. Kourlaba et al. [19]
Stroke, death within 30 days	3675	Government Gazette^†^ (DRG-code: N30Mα) & G. Kourlaba et al. [19]
Peripheral vascular disease, year of onset	5713	G. Kourlaba et al. [20]
Peripheral vascular disease, each subsequent year	1426	G. Kourlaba et al. [20]
**Direct costs of renal complications**		
Hemodialysis, first year	40352	D. Kaitelidou et al. & K. Souliotis et al. [17, 18]
Hemodialysis, each subsequent year	40352	Assumed equal with 1st year hemodialysis cost
Peritoneal dialysis, first year	46156	Experts opinion & Government Gazette^†^ (DRGs-code: Υ28Α and Y02M)
Peritoneal dialysis, each subsequent year	43656	Experts opinion & Government Gazette^†^ (DRG-code: Υ28Α)
Kidney transplant, first year	18489	S. Sidiropoulos [21]
Kidney transplant, each subsequent year	5400	K. Souliotis et al. [17]
**Direct costs of acute events**		
Major hypoglycaemic event	700	National School of Public Health (data on file)
Minor hypoglycaemic event	250	National School of Public Health (data on file)
**Direct costs of eye disease**		
Cost of laser treatment	731	Government Gazette† (DRG-code: O03A)
Cost of cataract operation	933	Government Gazette† (DRG-code: O15A)
Cost following cataract operation	59	Experts opinion & Drug price bulletin [15]
Cost of blindness in year of onset	11280	Experts opinion & K. Athanasakis et al. [16]
Cost of blindness in subsequent years	6200	Experts opinion & K. Athanasakis et al. [16]
**Direct costs of neuropathy, foot ulcer and amputation**		
Neuropathy, year of onset	1120	Experts opinion, Drug price bulletin [15] & Government Gazette^¶‡^
Neuropathy, each subsequent year	1120	Experts opinion, Drug price bulletin [15] & Government Gazette^¶‡^
Amputation, year of event	6629	Government Gazette† (DRG-code: K11M)
Amputation, prosthesis	1150	Government Gazette^‡^
Gangrene treatment	5282	Experts opinion, Drug price bulletin [15] & Government Gazette^†^ (DRGs-code: K11M & K13M)
Cost of infected ulcer treatment	2835	Experts opinion, Drug price bulletin [15] & Government Gazette^¶†^ (DRG-code: Δ20Μ)
Cost of uninfected ulcer treatment	387	Experts opinion, Drug price bulletin [15] & Government Gazette^¶^
Cost after healed ulcer	120	Experts opinion & Government Gazette^¶^
Cost of healed ulcer (history of amputation)	1471	Government Gazette† (DRG-code: Δ20Χ)
**Costs of interventions**		Drug price bulletin [15]
Liraglutide (1.2 mg)	3.44 per day	
Liraglutide (1.8 mg)	5.16 per day	
Sitagliptin	1.30 per day	
Exenatide BID	2.84 per day	
Metformin	0.11 per day	
Sulphonylurea (Glimepiride)	0.14 per day	
Basal insulin (Glargine)	1.38 per day	
**Consumable costs**		Government Gazette^#^
Needles for injectable drugs	0.17 per needle	
SMBG test strips	0.43 per strip	
SMBG lancets	0.09 per lancet	

*Reimbursement for screening tests (Ministerial Decree, Α4 /2878/4-6-1992).

^¶^Reimbursement for a doctor’s office visit (Presidential Decree, FEK 262A’/16-12-2011).

^†^Reimbursement for diagnosis-related groups (Common Ministerial Decree, FEK 946B’/27-3-2012).

^‡^EOPYY Health Provision Policy Regulation (Common Ministerial Decree, FEK 3054B’/18-11-2012).

^#^Reimbursement for consumables (Common Ministerial Decree, FEK 1561B’/21-06-2013).

ACE = Angiotensin-converting enzyme; SMBG = Self-monitoring blood glucose, DRG = Diagnosis-related group.

### Cost-effectiveness analysis

A non-parametric bootstrapping approach was employed for this health economic analysis. After running 1000 simulations over a cohort of 1000 non-identical patients generated with mean and standard deviation (SD) values, 1000 bootstrap samples were drawn. For each bootstrap iteration, the progression of diabetes was simulated in the patient cohort, to calculate mean (SD) costs, life expectancy and quality-adjusted life expectancy [10]. The results from the bootstrapped simulations were used to generate acceptability curves. Although there is no official willingness-to-pay threshold for Greece, a treatment was considered to be cost-effective at a threshold of €34000 per quality-adjusted life year (QALY) gained. This is based on the World Health Organisation (WHO) guidelines that state that a treatment should be considered cost-effective if the ICER is between 1 or 3 times the Gross Domestic Product (GDP) per capita of that country and a treatment is considered highly cost-effective at less than 1 times the GDP per capita [24]. Using current prices, the International Monetary Fund (IMF) estimated Greek GDP per capita at €17000 [25]. In the base case, quality-adjusted life expectancy and future costs were discounted at a rate of 3.5% annually which is the standard practice in Greece [26] and simulations were run over patients’ lifetimes.

### Sensitivity analyses

Univariate sensitivity analyses were performed for both treatment comparisons (1000 bootstrap iterations for each scenario) in order to evaluate the key drivers and robustness of the base case cost-effectiveness results. Specifically, individual parameters such as discount rate, cost of complications and treatment duration, were varied between low and high values within plausible ranges, whereas time horizon parameter was tested in a series of lower than base case values. In respect of parameters like disutility of major and minor hypoglycaemic events, values were removed or set all to one distinct value (−0.0052), as used in the National Institute for Health and Care Excellence (NICE) technology appraisal of insulin glargine [27]. For physiological parameters such as treatment effects in HbA1c, systolic blood pressure (SBP), blood lipids and BMI, the reported upper and lower 95% confidence interval (CI) in clinical trials for liraglutide arm were applied in the analyses. Further, the BMI benefit was assumed to be preserved after dropping incretin-based therapy. New parameters’ data were also tested via employing the United Kingdom Prospective Diabetes Study (UKPDS) progression equation for the first five years of the simulation or the Greek cohort characteristics instead of baseline patients’ characteristics from the clinical trials. Under the same rationale, as part of the follow-up to the 1860 clinical trial, one year clinical data for liraglutide vs. sitagliptin were also examined as well as a new treatment path involving a switch from sitagliptin to liraglutide after one year based on the 52- and 78-week extension studies, respectively [28, 29]. Last, in an attempt to compare exenatide with 1.2 mg liraglutide, we used the price of the 1.2 mg dose and ran scenarios with efficacy set to 100%, 90%, 80% and 70% of the 1.8 mg dose. This approach is based on observed values in LEAD1-4 studies [8], where the clinical effectiveness of liraglutide 1.2 mg found to be between 80-90% of the 1.8 mg dose, depending on the parameter in scope.

## Results

### Base case analyses

#### Liraglutide vs. sitagliptin

**Figure 1:**
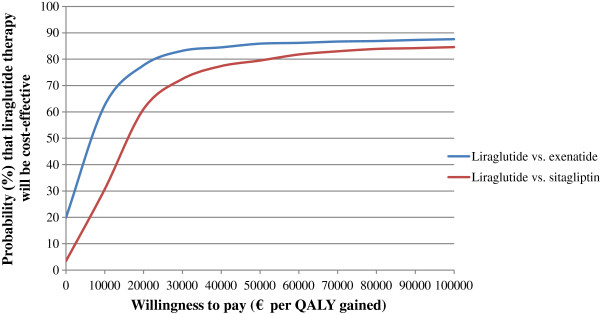
**Cost effectiveness acceptability curves of liraglutide vs. sitagliptin (red line) and liraglutide vs. exenatide (blue line).**

Compared with sitagliptin, liraglutide resulted in mean increases in discounted life expectancy and quality-adjusted life expectancy of 0.13 years (SD 0.23) and 0.19 QALYs (0.16) respectively, and was also correlated with greater lifetime health expenditures (€2797 [SD 1468]). Moreover, liraglutide was accompanied with lower cumulative incidence rates of long-term complications such as myocardial infarction, stroke and amputation (liraglutide: 17.16%, 8.79%, and 10.06% vs. sitagliptin: 17.83%, 9.24% and 10.54% respectively; Table 4). Considering both liraglutide and sitagliptin treatment arms, the largest component of lifetime direct costs was the drug acquisition costs, accounting for 38.13% versus 30.60% of total costs respectively, while the second largest comprised of the accrued ulcer, amputation and neuropathy costs (16.86% vs. 19.42% respectively). Based on the aforementioned health and cost outcomes, an ICER of €15101 per QALY gained was estimated for liraglutide (Table 5). The probability of liraglutide 1.2 mg being a cost-effective therapeutic option at a willingness-to-pay threshold of €30000 per QALY gained was above 70% (72.5%) (Figure 1).

**Table 4:** Cumulative incidence of long-term complications for liraglutide, sitagliptin and exenatide BID*

Complication	Liraglutide 1.2 mg	Sitagliptin	Difference	Liraglutide 1.8 mg	Exenatide BID	Difference
**Eye disease, %**						
Background retinopathy	17.18 (1.26)	19.26 (1.29)	−2.08	18.64 (1.24)	20.91 (1.27)	−2.27
Proliferative retinopathy	0.51 (0.22)	0.67 (0.27)	−0.15	0.61 (0.25)	0.78 (0.27)	−0.17
Severe vision loss	6.78 (0.76)	7.51 (0.83)	−0.73	6.59 (0.78)	7.46 (0.86)	−0.87
Macular edema	13.59 (1.11)	15.44 (1.14)	−1.85	12.95 (1.04)	14.88 (1.08)	−1.93
Cataract	10.62 (0.96)	11.11 (1.02)	−0.49	10.27 (1.02)	10.85 (1.05)	−0.58
**Renal disease, %**						
Microalbuminuria	24.22 (1.35)	27.02 (1.44)	−2.80	28.95 (1.43)	31.87 (1.55)	−2.92
Gross proteinuria	5.07 (0.71)	6.38 (0.78)	−1.30	6 (0.78)	7.26 (0.81)	−1.26
End-stage renal disease	0.58 (0.23)	0.83 (0.29)	−0.26	0.65 (0.26)	0.90 (0.3)	−0.26
**Diabetic foot & neuropathy, %**						
Foot ulcer	30.91 (1.42)	32.97 (1.48)	−2.07	28.36 (1.35)	30.21 (1.4)	−1.85
Amputation	10.06 (1.05)	10.54 (1.01)	−0.48	8.63 (0.98)	9.13 (1.01)	−0.51
Neuropathy	47.51 (1.75)	52.27 (1.69)	−4.76	45.74 (1.61)	50.13 (1.71)	−4.39
**Cardiovascular disease, %**						
Congestive heart failure	13.23 (1.05)	14.29 (1.08)	−1.06	13.54 (1.09)	14.31 (1.15)	−0.78
Peripheral vascular disease	9.34 (0.90)	10.82 (1)	−1.49	8.69 (0.88)	10.43 (0.94)	−1.73
Angina	13.01 (1.11)	13.52 (1.11)	−0.51	12.05 (1.02)	12.87 (1.08)	−0.82
Stroke	8.79 (0.95)	9.24 (0.91)	−0.46	8.69 (0.94)	9.45 (0.92)	−0.76
Myocardial infarction	17.16 (1.19)	17.83 (1.22)	−0.68	16.31 (1.17)	17.55 (1.25)	−1.24
**Hypoglycaemia, %**						
Major hypoglycaemia	0.11 (0.01)	0 (0)	0.11	0 (0)	0.22 (0.02)	−0.22
Minor hypoglycaemia	23.63 (0.48)	22.88 (0.45)	0.75	31.66 (0.50)	34.49 (0.46)	−2.84

*Values shown are mean (SD) cumulative incidences over patient lifetimes from the base case modelling simulation expressed as a percentage of patients experiencing events.

**Table 5:** **Health and economic outcomes (ICERs) of the base case analyses**

	Liraglutide 1.2 mg	Sitagliptin	Difference
Discounted life expectancy (years)	14.22 (0.18)	14.09 (0.17)	0.13 (0.23)
Discounted quality-adjusted life expectancy (QALYs)	9.24 (0.12)	9.05 (0.11)	0.19 (0.16)
Discounted total lifetime direct medical costs (€)	39524 (1100)	36727 (1113)	2797 (1468)
Drug acquisition	15069	11240	3829
Patient management*	1939	1928	11
Cardiovascular disease	5858	6170	−312
Renal disease	1146	1253	−107
Diabetic foot and neuropathy	6665	7132	−467
Eye disease	5325	5676	−351
Hypoglycaemia	3522	3329	193
ICER (€ per QALY gained)			15101
	**Liraglutide 1.8 mg**	**Exenatide BID**	**Difference**
Discounted life expectancy (years)	14.10 (0.17)	13.96 (0.16)	0.14 (0.23)
Discounted quality-adjusted life expectancy (QALYs)	9.22 (0.12)	9.03 (0.11)	0.19 (0.16)
Discounted total lifetime direct medical costs (€)	43236 (1049)	41934 (1065)	1302 (1492)
Drug acquisition	18505	14927	3578
Patient management*	1931	1920	11
Cardiovascular disease	5544	5980	−436
Renal disease	1611	1692	−81
Diabetic foot and neuropathy	5859	6317	−458
Eye disease	4461	4948	−487
Hypoglycaemia	5326	6149	−823
ICER (€ per QALY gained)			6818

Values shown are means with SDs in parentheses. *Concomitant medication and screening. QALY = quality-adjusted life year; € = 2013 Euros; ICER = incremental cost-effectiveness ratio.

#### Liraglutide vs. exenatide

In comparison with exenatide, mean increases in discounted life expectancy and quality-adjusted life expectancy of 0.14 years (SD 0.23) and 0.19 QALYs (0.16) were associated with liraglutide 1.8 mg over a patient’s lifetime. These clinical benefits were endorsed by the reduced cumulative incidence of diabetes-related complications noted in the liraglutide arm (Table 4). Relating to the lifetime direct costs, liraglutide maintained a more costly profile and was connected with higher mean costs of €1302 (SD 1492) with major cost contributor being again the drug acquisition costs (proportion of total costs: 42.80% for liraglutide vs. 35.60% for exenatide). The second most significant cost driver remained the costs related to the diabetic foot and neuropathy complications (13.55% for liraglutide vs. 35.60% for exenatide). The ICER of liraglutide versus exenatide was estimated at €6818 per QALY gained (Table 5). At a willingness-to-pay threshold of €30000 per QALY gained, liraglutide was found to be the accepted intervention in over 83.2% of cases (Figure 1).

### Sensitivity analyses

#### Liraglutide vs. sitagliptin and liraglutide vs. exenatide

As depicted in Table 6, simulation results were quite sensitive to the gradual shortening of model time horizon resulting in an increase of base case ICER for liraglutide 1.2 mg by more than 600% at 5 years simulation. Nevertheless, it should be stressed that liraglutide maintained its cost-effective profile when time horizon was set to 30 and 20 years under a willingness-to-pay of €34000 per QALY gained. Setting the discount rate to 0 a lower ICER was traced in favour of liraglutide 1.2 mg, while in the opposite direction of increasing it to 6% the corresponding ICER augmented. In the sensitivity scenario of rising or diminishing the base case values of all complication costs by 10 percent, no major changes in ICERs of liraglutide 1.2 mg were observed. On the contrary, simulation results were quite sensitive to patients’ HbA1c values underlining, as such, the importance of this biochemical parameter to health and cost outcomes of model analysis. In fact, the upper and lower 95% CI limits of the liraglutide 1.2 mg effect in HbA1c were associated with quite higher and lower ICERs respectively. When HbA1c was assumed to follow the UKPDS creep, the respective ICER grew more than two times (117%) since the HbA1c benefit of liraglutide 1.2 mg was not preserved. Changes in other physiological measures, as well as utility decrements attached to hypoglycaemic events, had leaner impact on the liraglutide 1.2 mg ICERs. In the Greek cohort analysis, although a quite elevated ICER (increased by 61.3%) was traced, yet liraglutide 1.2 mg continued to be a cost-effective option at the defined willingness-to-pay threshold. Likewise, when we varied the length of liraglutide treatment between 3 and 7 years towards replicating a real-life setting the cost-effective profile of liraglutide treatment was retained. 52-week clinical data were associated with an ICER reduced by 30%, while switching patients to liraglutide after 1 year of sitagliptin treatment demonstrated a greater ICER noting a 32.2% increase. Last, comparing liraglutide 1.2 mg with exenatide showed that the liraglutide treatment is the dominant strategy in all efficacy scenarios. As regards liraglutide 1.8 mg, similar trends were found (Table 6).

**Table 6:** **Summary of results of the sensitivity analyses**

Sensitivity analysis	Liraglutide 1.2 mg vs. sitagliptin			Liraglutide 1.8 mg vs. exenatide BID		
	Difference in QALYs	Difference in costs [€]	ICER [€ per QALY gained]	Difference in QALYs	Difference in costs [€]	ICER [€ per QALY gained]
***Base case***	*0.19 (0.16)*	*2797 (1468)*	*15101*	*0.19 (0.16)*	*1302 (1492)*	*6818*
**Model time horizon:**						
30 years	0.18 (0.15)	2857 (1428)	15767	0.18 (0.15)	1380 (1541)	7874
20 years	0.12 (0.11)	2946 (1186)	23797	0.12 (0.12)	1531 (1218)	12531
10 years	0.06 (0.06)	3569 (791)	61595	0.05 (0.06)	2306 (774)	43977
5 years	0.04 (0.02)	3787 (452)	106618	0.03 (0.03)	2458 (516)	84503
**Discount rate:**						
0%	0.34 (0.31)	2319 (2759)	6776	0.36 (0.30)	552 (2767)	1529
6%	0.13 (0.11)	2879 (1057)	22353	0.13 (0.11)	1529 (1081)	11754
**Costs of complications:**						
Increased by 10%	0.19 (0.16)	2694 (1605)	14541	0.19 (0.16)	1074 (1630)	5625
Decreased by 10%	0.19 (0.16)	2902 (1323)	15666	0.19 (0.16)	1531 (1355)	8020
**Physiological parameters:**						
HbA1c 95% upper limit	0.23 (0.15)	2411 (1565)	10334	0.26 (0.16)	798 (1500)	3101
HbA1c 95% lower limit	0.14 (0.16)	3270 (1557)	23725	0.12 (0.16)	1947 (1500)	15961
UKPDS creep of HbA1c for 5 years	0.10 (0.15)	3362 (1597)	32756	0.10 (0.15)	1922 (1613)	18940
SBP 95% upper limit	0.20 (0.16)	2784 (1517)	14086	0.21 (0.15)	1142 (1500)	5380
SBP 95% lower limit	0.18 (0.15)	2897 (1468)	16246	0.19 (0.15)	1375 (1444)	7327
Lipid 95% upper limit	0.22 (0.16)	2744 (1541)	12693	0.22 (0.15)	1162 (1605)	5373
Lipid 95% lower limit	0.17 (0.16)	2781 (1492)	16577	0.16 (0.15)	1459 (1525)	9400
BMI 95% upper limit	0.20 (0.15)	2810 (1500)	13823	0.20 (0.17)	1274 (1484)	6231
BMI 95% lower limit	0.18 (0.16)	2820 (1500)	16030	0.18 (0.16)	1269 (1460)	7263
BMI benefit maintained	0.25 (0.15)	2759 (1476)	11105	0.21 (0.16)	1312 (1492)	6408
**Hypoglycaemia:**						
No hypo disutility	0.19 (0.16)	2797 (1468)	14867	0.18 (0.16)	1302 (1492)	7272
Disutility of −0.0052 for all hypos	0.19 (0.16)	2797 (1468)	15118	0.19 (0.16)	1302 (1492)	6699
**Treatment duration:**						
7 years	0.20 (0.15)	3941 (1428)	19773	0.20 (0.17)	2114 (1557)	10458
3 years	0.19 (0.17)	1329 (1500)	7123	0.19 (0.16)	275 (1460)	1480
**Greek cohort data**	0.12 (0.14)	3008 (1775)	24355	0.12 (0.15)	1586 (1928)	13424
**52 week clinical data**	0.22 (0.17)	2309 (1508)	10564			
**Liraglutide delayed by 1 year**	0.12 (0.16)	2340 (1581)	19960	-	-	-
**Liraglutide 1.2 mg cost &:**						
100% of 1.8 mg efficacy	-	-	-	0.19 (0.16)	−1473 (1492)	Lira. Dominant
90% of 1.8 mg efficacy	-	-	-	0.14 (0.15)	−1016 (1500)	Lira. Dominant
80% of 1.8 mg efficacy	-	-	-	0.08 (0.15)	−625 (1613)	Lira. Dominant
70% of 1.8 mg efficacy	-	-	-	0.02 (0.15)	−126 (1525)	Lira. Dominant

Data are mean (SD). QALY = quality-adjusted life year; € = 2013 Euros; ICER = incremental cost-effectiveness ratio; HbA1c = glycated haemoglobin; UKPDS = United Kingdom Prospective Diabetes Study; SBP = systolic blood pressure; BMI = body mass index; Hypo = hypoglycaemic event; Lira = Liraglutide.

## Discussion

Long-term projections suggested that once daily doses of 1.2 and 1.8 mg of liraglutide treatment result in greater improvements in life-expectancy and quality-adjusted life expectancy, as well as reduced incidence rates of diabetes-related complications compared with once daily 100 mg sitagliptin or twice daily 10 μg exenatide, respectively. From a third-party perspective (EOPYY), evaluation of cost-effectiveness based on quality-adjusted life expectancy of 1.8 and 1.2 mg liraglutide doses generated ICERs varying from €6818 to €15101 per QALY gained, respectively. These ICERs, in fact, lie below the threshold of €34000 per QALY gained and, thus, liraglutide 1.2 mg combined with metformin monotherapy and liraglutide 1.8 mg combined with metformin and sulphonylurea are found to be highly cost-effective strategies for the treatment of T2DM versus sitagliptin or exenatide, respectively. In the base case analysis, liraglutide treatment was associated with higher direct medical costs over patients’ lifetime, mainly due to increased drug acquisition costs. However, these were partially curbed by the cost savings marked in the treatment of diabetes-related complications due to the lower risk of unfolding these complications with liraglutide therapy.

In various country-specific settings, earlier published cost-effectiveness analyses have been focused on comparing liraglutide with sitagliptin or exenatide. Based on the results of the present analysis, we reach similar conclusions as these previous evaluations where liraglutide was demonstrated to be a cost-effective strategy, under the reported willingness-to-pay thresholds, from a healthcare payer perspective. In specific, regarding the liraglutide 1.2 mg versus sitagliptin comparison, Lee et al. [30] calculated an ICER of $25742/QALY gained for the former treatment in the United States (US), while in the United Kingdom Davies et al. [23] found the subcutaneous treatment to be associated with an ICER of £9851/QALY gained. Similarly, in Spain [31] liraglutide 1.2 mg resulted in an ICER of €13266/QALY gained. Considering the liraglutide 1.8 mg versus exenatide (BID) comparison, the former treatment exhibited an ICER of $40282/QALY gained in the US payer setting [32], while in the European Union setting (in six different countries: Switzerland, Denmark, Norway, Finland, The Netherlands, and Austria) liraglutide was associated with ICERs ranged between €6902 and €13546/QALY gained [33]. Overall, the results across the various country-specific settings are in line with results reported here and no meaningful deviations were traced.

In general, the sensitivity analyses indicated that these results were robust under a range of assumptions. In the liraglutide against sitagliptin comparison, the key drivers of cost-effectiveness were HbA1c and weight (to a lesser extent), with only small impact from the rest of the physiological parameters. These findings, are in accordance with previous cost-effectiveness studies [23, 31], as well as the higher efficacy of liraglutide versus sitagliptin in decreasing HbA1c and weight reported in the 1860 clinical study. In to the liraglutide against exenatide comparison, HbA1c was the most important driver of cost-effectiveness in line with previous literature [32, 33], with only small effects from the other physiological parameters. This is a quite reassuring remark, since the HbA1c effect was the primary efficacy endpoint of the LEAD 6 trial and benefit in HbA1c related with liraglutide was shown to be significantly greater than that observed with exenatide. In light of both treatment comparisons, remarkably lower ICERs were yielded in case of 3 year treatment duration, as the accumulated effectiveness of each therapy reached almost at the same level of the base case, whereas the respective direct total costs incurred by patients were significantly lower given the shortening of drugs usage duration. This outcome, indeed, is quite interesting and may be in need of further investigation at a later date. In respect of time horizon assumptions, the cost-effectiveness results were quite sensitive (upward trend) to deviations from baseline model timeframe, most probably on grounds of the considerable lag time in the manifestation of diabetes complications after long periods of poor glycaemic control. The shortening of simulation time horizons to this extent halters the emergence of any relative differences in complication rates. Additionally, when Greek cohort data geared higher ICERs were estimated. This could be attributed to the fact that the Greek intervention cohort might have been in a more advanced stage of disease progression, since the mean age and duration of diabetes were significantly higher than in the 1860 and LEAD-6 trial cohorts, and so did some mean baseline biochemical risk characteristics and proportions with comorbidity complications (Table 1). In this respect, the aging process alongside with the overall worse baseline cohort features likely had more severe consequences in patients’ health during model simulations, resulting, thus, in much lower accumulated QALYs considering all treatment comparators. Nonetheless, it should be stressed that the ICER values were found to be below the threshold of €34000 per QALY gained and, therefore, liraglutide treatment continued to be a cost-effective option. Furthermore, delaying liraglutide 1.2 mg treatment by 1 year was an unfavourable option (increased ICER) similar to findings in a previous study carried out in Spain [31]. The displayed liraglutide 1.2 mg dominance over exenatide was expected given the current clinical and cost data.

Several potential limitations to this study should be considered. First, the generalizability of the present findings is constrained to those patients with relatively advanced disease (Table 1), who were insufficiently controlled with OADs. Moreover, based on three recent studies [34–36] and clinical experts opinion it was assumed that HbA1c could be maintained over time with adequate pharmacotherapy, in both insulin and non-insulin users, in contrast to the UKPDS progression equation according to which, HbA1c levels gradually increase as beta-cell function diminishes over time [10]. In this regard, the results of the present study are fully related to this assumption in an attempt to best reflect current clinical practice. Additionally, like all models used to gauge the long-term outcomes of patients with T2DM, this model makes long-term projections of outcomes relied upon the findings of short-term studies. Nevertheless, the CDM has been validated against real life data demonstrating a reliable predicting analysis behaviour [9].

## Conclusions

To conclude, the present economic evaluation suggests that, under a willingness-to-pay threshold of €34000 per QALY gained, liraglutide was estimated to be a highly cost-effective option for the treatment of T2DM in a Greek setting.

### Compliance with ethics

This study is an economic evaluation analysis and does not involve human subjects. Input data including human material or human data derived from other published studies performed with the approval of an appropriate ethics committee. Therefore, no ethics approval arises for the performance of this cost-effectiveness analysis.
